# Calcium Hippurate in Dyspesia, Diabetis, Etc.

**Published:** 1886-04-20

**Authors:** 


### CALCIUM HIPPURATE IN DYSPEPSIA, DIABETES, ETC.

The hippurate is especially useful in inflammatory dyspepsias, either gastric or intestinal, with flatulence, vertigo, and migraine. As a tonic to the digestive organs, it is far superior to any of the vegetable remedies. It is indicated not only where the gastric secretion is more abundant than normal, but also, where it is diminished in quantity. Far from producing constipation, the hippurate hastens alvine evacuations. and, alone, is sufficient to combat the stubborn constipation that accompanies certain dyspepsias. Inversely, it controls diarrhœa due to indigestion ; and is especially advantageous in gouty subjects and lymphatic individuals. A circumstance worthy of remark here, is that both the gouty and the lymphatic excrete an excess of uric acid and the urates, and sometimes, a superabundance of the phosphates.

Scrofulides of the mucous membranes are also successfully treated by the hippurate. It acts admirably, too, in gastro-intestinal acidity, and by its corrective action on the function of the liver, aids materially in diminishing the amount of sugar excreted in diabetes.

### GOUTY RHETMATISM, GOUT, ETC.

It is generally admitted that in gouty rheumatism, the urine contains an excess of uric acid. In ratchism, osteomalachia and in certain cases of non-uniting fractures, the urine is habitually too rich in nitrates and phosphates. In fine, lime and the phosphates appear to be needed by children who suffer from slow dentition. In all these cases, then, the hippurate of lime is a very useful remedy. Instead of administering it alone, however, it may be aided by small doses of phosphate of soda at meal times.

The following is Poulet's formula for "syrup of hippurate of lime:"

℞. Hippuric acid, pure .................. 100. grammes,
Milk of lime (whiting size) ....q. s. to produce an alkaline reaction,
Hot water .............................. 2 litres,
Sugar ................................. 2400 grammes,
Essence of Citron ..................... 15 grammes.

Dissolve the hippuric acid in a portion of the hot water (80° centig.), and stir in the whiting size (*lait de chaux*) *gradually* until litmus paper gives us an alkaline reaction. To do this properly requires at least a quarter of an hour's work. Then add the rest and place over a slow fire, etc. Dose : A tablespoonful three or four times a day.—*Investigator.*

